# Supplementary figures and images for: Intra-individual heteroplasmy in the Gentiana tongolensis plastid genome (Gentianaceae)
Source: PeerJ. 2019 Nov 27;7:e8025. doi: 10.7717/peerj.8025 (PMC6884991; doi:10.7717/peerj.8025)

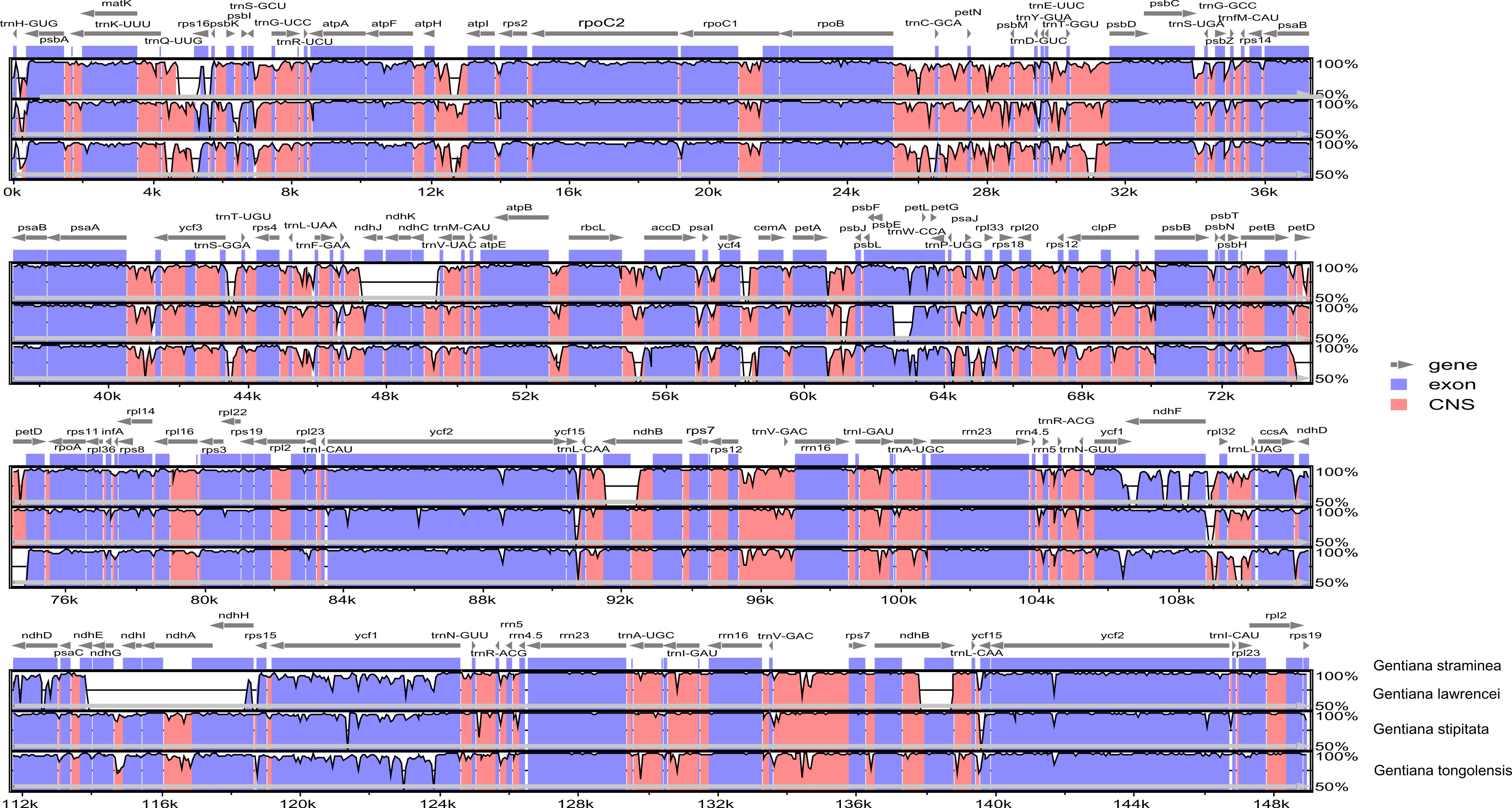

Supplement: Supplemental Information 1 [file peerj-07-8025-s001.jpg]
