# Supplementary material for: Intra-individual heteroplasmy in the Gentiana tongolensis plastid genome (Gentianaceae)
Source: PeerJ. 2019 Nov 27;7:e8025. doi: 10.7717/peerj.8025 (PMC6884991; doi:10.7717/peerj.8025)
Supplement: Supplemental Information 2 [file peerj-07-8025-s002.docx]

| Name | Sequence (5'-3') |
| --- | --- |
| GTcpSNP1 | F: CATCATGCCCCTTATGCCCT |
|  | R: TTTACCTCTTTCGCCCCCAC |
| GTcpSNP2 | F: GGTAAAAGAAGTTCACGACC |
|  | R: AATGAGAATGGATAAGAGGC |
| GTcpSNP3 | F: ATAATCGTAACCTGGTCCCG |
|  | R: ATGCTAGTTTTAACAACTCC |
| GTcpSNP4 | F: GTCCGTATCGGTAGAAACAG |
|  | R: TTCATGGGAGAAAATTGAGT |
| GTcpSNP5 | F: TCTGGCATTGGGTGTATTGG |
|  | R: CAGGCTTCTGCTAAACTTTGATTT |
