# Supplementary material for: Intra-individual heteroplasmy in the Gentiana tongolensis plastid genome (Gentianaceae)
Source: PeerJ. 2019 Nov 27;7:e8025. doi: 10.7717/peerj.8025 (PMC6884991; doi:10.7717/peerj.8025)
Supplement: Supplemental Information 3 [file peerj-07-8025-s003.docx]

| Position | Reference Base | SNP | Allelic depths of Reference Base | Frequency of SNP | Location | Region |
| --- | --- | --- | --- | --- | --- | --- |
| 14 | A | G | 2843 | 0.31 | trnH | LSC |
| 30 | A | G | 3265 | 0.297 | trnH | LSC |
| 54 | A | G | 3794 | 0.281 | trnH | LSC |
| 8829 | G | A | 3652 | 0.132 | atpA | LSC |
| 8848 | GATCATCA | G | 3550 | 0.111 | atpA | LSC |
| 8861 | A | G | 3621 | 0.126 | atpA | LSC |
| 8975 | A | C | 3756 | 0.122 | atpA | LSC |
| 8984 | T | C | 3669 | 0.12 | atpA | LSC |
| 9020 | C | T | 3659 | 0.125 | atpA | LSC |
| 9063 | A | C | 3627 | 0.126 | atpA | LSC |
| 9086 | G | A | 3651 | 0.144 | atpA | LSC |
| 9119 | T | C | 3617 | 0.137 | atpA | LSC |
| 9137 | T | G | 3701 | 0.139 | atpA | LSC |
| 9142 | C | A | 3700 | 0.14 | atpA | LSC |
| 9174 | TCAATAG | T | 3637 | 0.118 | atpA | LSC |
| 9206 | A | G | 3634 | 0.115 | atpA | LSC |
| 9214 | A | G | 3554 | 0.112 | atpA | LSC |
| 15515 | A | G | 3341 | 0.024 | rpoC2 | LSC |
| 16631 | C | A | 2979 | 0.026 | rpoC2 | LSC |
| 21318 | A | C | 3645 | 0.11 | rpoC1 exon 2 | LSC |
| 21366 | C | A | 3694 | 0.125 | rpoC1 exon 2 | LSC |
| 21378 | C | T | 3711 | 0.129 | rpoC1 exon 2 | LSC |
| 21397 | T | C | 3635 | 0.12 | rpoC1 exon 2 | LSC |
| 21778 | G | A | 5094 | 0.021 | rpoB | LSC |
| 21779 | C | A | 5114 | 0.031 | rpoB | LSC |
| 21796 | G | A | 5345 | 0.03 | rpoB | LSC |
| 21802 | C | T | 5315 | 0.046 | rpoB | LSC |
| 21804 | C | T | 5315 | 0.064 | rpoB | LSC |
| 21805 | C | T | 5308 | 0.029 | rpoB | LSC |
| 21806 | A | G | 5287 | 0.027 | rpoB | LSC |
| 21811 | C | A | 5250 | 0.03 | rpoB | LSC |
| 21811 | C | T | 5250 | 0.04 | rpoB | LSC |
| 21814 | C | T | 5239 | 0.037 | rpoB | LSC |
| 21819 | G | A | 5153 | 0.023 | rpoB | LSC |
| 21821 | C | T | 5123 | 0.084 | rpoB | LSC |
| 21822 | C | T | 5116 | 0.022 | rpoB | LSC |
| 21827 | C | A | 4985 | 0.053 | rpoB | LSC |
| 21827 | C | T | 4985 | 0.032 | rpoB | LSC |
| 21830 | C | A | 4909 | 0.021 | rpoB | LSC |
| 21830 | C | T | 4909 | 0.036 | rpoB | LSC |
| 21948 | T | TTAAAAA | 3512 | 0.11 | rpoB | LSC |
| 22052 | C | T | 3783 | 0.144 | rpoB | LSC |
| 22169 | C | T | 3841 | 0.136 | rpoB | LSC |
| 22457 | A | G | 3518 | 0.149 | rpoB | LSC |
| 22574 | A | C | 3402 | 0.135 | rpoB | LSC |
| 22711 | T | C | 3549 | 0.127 | rpoB | LSC |
| 22847 | A | G | 3475 | 0.136 | rpoB | LSC |
| 22888 | G | C | 3489 | 0.131 | rpoB | LSC |
| 23192 | A | G | 3545 | 0.155 | rpoB | LSC |
| 23411 | C | T | 3586 | 0.145 | rpoB | LSC |
| 25234 | T | TA | 3100 | 0.043 | rpoB-trnC | LSC |
| 25234 | TA | T | 4481 | 0.031 | rpoB-trnC | LSC |
| 26294 | T | C | 5040 | 0.068 | trnC-petN | LSC |
| 27625 | C | T | 3463 | 0.024 | petN-psbM | LSC |
| 28066 | C | CT | 3142 | 0.029 | psbM-trnD | LSC |
| 28066 | CT | C | 3223 | 0.033 | psbM-trnD | LSC |
| 30628 | A | G | 3021 | 0.029 | psbD | LSC |
| 31648 | CTCTAGGTT | C | 3353 | 0.105 | psbC | LSC |
| 32024 | G | A | 3374 | 0.138 | psbC | LSC |
| 32108 | C | G | 3608 | 0.112 | psbC | LSC |
| 32890 | G | T | 3372 | 0.063 | psbC-trnS | LSC |
| 32891 | A | C | 3363 | 0.063 | psbC-trnS | LSC |
| 34736 | C | T | 3166 | 0.024 | rps14-psaB | LSC |
| 38204 | C | T | 3712 | 0.113 | psaA | LSC |
| 38948 | T | G | 3792 | 0.184 | psaA | LSC |
| 39182 | A | G | 3343 | 0.031 | psaA | LSC |
| 41331 | C | A | 3418 | 0.029 | ycf3 intron | LSC |
| 42318 | T | C | 3584 | 0.042 | ycf3-trnS | LSC |
| 42472 | G | A | 3462 | 0.051 | trnS-GGA | LSC |
| 43934 | G | T | 3531 | 0.046 | trnT-trnL | LSC |
| 45843 | CT | C | 3709 | 0.025 | trnF-ndhJ | LSC |
| 47895 | A | T | 3558 | 0.03 | ndhC-trnM | LSC |
| 48034 | A | G | 3376 | 0.026 | ndhC-trnM | LSC |
| 48049 | CT | C | 3384 | 0.031 | ndhC-trnM | LSC |
| 48218 | G | T | 3481 | 0.043 | ndhC-trnM | LSC |
| 48789 | C | CTTATTGCT | 3247 | 0.027 | ndhC-trnM | LSC |
| 50365 | C | A | 3371 | 0.026 | atpB | LSC |
| 51439 | C | A | 3526 | 0.033 | atpB-rbcL | LSC |
| 53389 | C | A | 3582 | 0.025 | rbcL-accD | LSC |
| 53611 | G | GA | 3247 | 0.031 | rbcL-accD | LSC |
| 53611 | GA | G | 3506 | 0.039 | rbcL-accD | LSC |
| 53624 | A | C | 3544 | 0.035 | rbcL-accD | LSC |
| 54355 | G | A | 3347 | 0.034 | accD | LSC |
| 55398 | T | C | 3231 | 0.025 | accD-psaI | LSC |
| 56268 | G | C | 3366 | 0.03 | psaI-ycf4 | LSC |
| 57863 | ATAAAAT | A | 3535 | 0.034 | cemA-petA | LSC |
| 58755 | A | G | 3334 | 0.042 | petA | LSC |
| 59136 | T | TTTTCTACC | 3365 | 0.023 | petA-psbJ | LSC |
| 59271 | A | G | 3491 | 0.042 | petA-psbJ | LSC |
| 59299 | TA | T | 3542 | 0.024 | petA-psbJ | LSC |
| 59576 | T | A | 3394 | 0.036 | petA-psbJ | LSC |
| 60037 | A | C | 3507 | 0.037 | psbJ-psbL | LSC |
| 61053 | A | G | 3447 | 0.03 | psbE-petL | LSC |
| 61235 | T | G | 3323 | 0.038 | psbE-petL | LSC |
| 61603 | TA | T | 3426 | 0.027 | psbE-petL | LSC |
| 61676 | G | A | 3418 | 0.027 | petL | LSC |
| 61824 | T | C | 3179 | 0.03 | petL-petG | LSC |
| 62069 | G | A | 3438 | 0.075 | petG-trnW | LSC |
| 62073 | C | A | 3399 | 0.076 | petG-trnW | LSC |
| 62121 | T | G | 3556 | 0.119 | petG-trnW | LSC |
| 65033 | C | T | 3332 | 0.051 | rpl20-rps12 | LSC |
| 65105 | TA | T | 3341 | 0.023 | rpl20-rps12 | LSC |
| 66799 | A | G | 3364 | 0.024 | clpP intron 1 | LSC |
| 67233 | T | C | 3237 | 0.023 | clpP intron 1 | LSC |
| 68370 | TTCTTTGGATTTACCCAAGA | T | 3635 | 0.097 | psbB | LSC |
| 68436 | C | T | 3866 | 0.169 | psbB | LSC |
| 68516 | CTGTAAA | C | 4405 | 0.255 | psbB | LSC |
| 68569 | A | G | 3998 | 0.171 | psbB | LSC |
| 68593 | G | A | 4360 | 0.092 | psbB | LSC |
| 68595 | T | G | 4324 | 0.093 | psbB | LSC |
| 68616 | T | A | 4296 | 0.089 | psbB | LSC |
| 68769 | GATTGAATT | G | 3672 | 0.153 | psbB | LSC |
| 68943 | G | A | 3819 | 0.201 | psbB | LSC |
| 69026 | G | A | 3780 | 0.2 | psbB | LSC |
| 70158 | C | T | 3369 | 0.027 | psbH | LSC |
| 70637 | A | T | 3249 | 0.025 | psbH-petB | LSC |
| 72343 | A | G | 3397 | 0.025 | petD | LSC |
| 72765 | T | C | 3321 | 0.035 | rpoA | LSC |
| 73400 | G | T | 3568 | 0.029 | rpoA | LSC |
| 73653 | T | C | 3948 | 0.131 | rpoA | LSC |
| 74040 | T | C | 3047 | 0.028 | rps11 | LSC |
| 103791 | T | C | 3350 | 0.035 | ndhF | SSC |
| 103983 | C | T | 3489 | 0.024 | ndhF | SSC |
| 104979 | T | C | 3157 | 0.03 | ndhF | SSC |
| 105100 | A | G | 3182 | 0.037 | ndhF | SSC |
| 105346 | A | G | 3414 | 0.023 | ndhF | SSC |
| 105403 | T | C | 3298 | 0.039 | ndhF | SSC |
| 105518 | A | G | 3276 | 0.029 | ndhF | SSC |
| 105998 | T | C | 3319 | 0.033 | ndhF-rpl32 | SSC |
| 106586 | G | A | 2926 | 0.024 | rpl32-trnL | SSC |
| 106797 | G | C | 3237 | 0.038 | rpl32-trnL | SSC |
| 107885 | T | TA | 3407 | 0.026 | ccsA | SSC |
| 107885 | TA | T | 3689 | 0.024 | ccsA | SSC |
| 108379 | T | A | 3270 | 0.025 | ccsA-ndhD | SSC |
| 111701 | C | CA | 3073 | 0.028 | ndh-ndhI | SSC |
| 111701 | CA | C | 3324 | 0.029 | ndh-ndhI | SSC |
| 116061 | C | T | 3412 | 0.028 | ycf1 | SSC |
| 116721 | G | GT | 3402 | 0.036 | ycf1 | SSC |
| 116721 | GT | G | 3466 | 0.035 | ycf1 | SSC |
| 117278 | G | GT | 3003 | 0.045 | ycf1 | SSC |
| 118279 | G | GT | 3355 | 0.03 | ycf1 | SSC |
| 120290 | C | CT | 3422 | 0.029 | ycf1 | SSC |
| 120290 | CT | C | 3479 | 0.026 | ycf1 | SSC |
