# Supplementary material for: Intra-individual heteroplasmy in the Gentiana tongolensis plastid genome (Gentianaceae)
Source: PeerJ. 2019 Nov 27;7:e8025. doi: 10.7717/peerj.8025 (PMC6884991; doi:10.7717/peerj.8025)
Supplement: Supplemental Information 4 — Dots represent the first nucleotide in each column. Dashes represent deletion. Results of the three individuals are represented in purple, black and red color, respectively. The individual used for Illumina sequencing is the red one. [file peerj-07-8025-s004.docx]

| Position | ## | ## | ## | ## | 25 | 73 | ## | ## | ## | 199 | ## | ## | ## | ## | ## | ## | ## | ## | ## | ## | ## | ## | ## | ## | ## | ## | ## | ## | ## | ## | ## | ## | ## | ## | ## | ## | ## | ## | ## |
| --- | --- | --- | --- | --- | --- | --- | --- | --- | --- | --- | --- | --- | --- | --- | --- | --- | --- | --- | --- | --- | --- | --- | --- | --- | --- | --- | --- | --- | --- | --- | --- | --- | --- | --- | --- | --- | --- | --- | --- |
| Plastome | C | T | A | T | C | G | T | T | - | TTT | T | A | G | T | A | A | A | - | A | T | G | T | A | C | A | A | A | T | T | A | G | T | A | T | - | T | T | A | A |
| Hap_1 | . | . | . | . | . | . | . | . | T | … | . | . | . | . | . | . | . | . | G | . | . | . | . | . | . | . | . | . | . | . | . | . | . | . | . | . | . | . | . |
| Hap_2 | . | . | . | . | . | . | . | . | . | … | . | . | . | . | . | . | - | . | . | . | . | . | . | . | . | . | . | . | . | . | . | . | . | . | . | C | . | . | . |
| Hap_4 | . | . | . | . | . | . | . | . | . | … | . | . | . | . | . | . | . | . | . | . | . | . | . | . | . | . | . | . | . | . | . | C | G | . | . | . | . | . | . |
| Hap_5 | . | . | . | . | . | . | . | . | . | … | . | G | . | C | . | . | . | . | . | . | . | . | . | . | . | . | . | . | . | . | . | . | . | . | . | . | . | . | . |
| Hap_6 | . | . | . | . | . | . | . | . | . | --- | . | . | . | . | . | . | . | . | . | . | . | . | . | . | . | . | . | . | . | . | . | . | . | . | . | . | . | . | . |
| Hap_7 | . | . | . | . | . | . | . | . | . | … | . | . | . | . | . | . | . | . | . | . | . | . | . | . | . | . | . | . | . | . | . | . | . | . | G | . | . | . | . |
| Hap_8 | . | . | . | . | . | . | . | . | . | … | . | . | . | . | . | . | . | . | . | . | . | . | . | . | . | G | . | . | . | . | . | . | . | . | . | . | . | . | . |
| Hap_9 | . | . | . | . | . | . | . | . | . | … | . | . | . | . | . | . | . | . | . | . | . | . | . | T | . | . | . | . | . | . | . | . | . | . | . | . | . | . | . |
| Hap_10 | . | . | . | . | . | . | . | . | . | … | . | . | . | . | . | . | . | . | . | . | . | . | . | . | . | . | . | . | . | G | . | . | . | . | . | . | . | . | . |
| Hap_11 | . | . | . | . | T | . | . | . | . | … | . | . | . | . | . | . | . | . | . | . | . | . | . | . | . | . | . | . | . | . | . | . | . | . | . | . | . | . | . |
| Hap_12 | . | . | . | . | . | . | . | . | . | … | . | . | . | . | . | . | . | . | . | . | . | . | . | . | . | . | G | . | . | . | . | . | . | . | . | . | . | - | . |
| Hap_13 | . | . | . | . | . | . | . | . | . | … | . | . | . | . | . | . | . | . | . | . | . | . | . | . | . | . | . | . | . | . | A | . | . | . | . | . | . | . | . |
| Hap_14 | . | . | G | . | . | . | . | . | . | … | . | . | . | . | . | . | . | . | . | . | . | . | . | . | . | . | . | . | . | . | . | . | . | . | . | . | . | . | . |
| Hap_15 | . | C | . | . | . | . | . | . | . | … | . | . | . | . | . | . | . | . | . | . | . | . | . | . | . | . | . | . | . | . | . | . | . | . | . | . | . | . | . |
| Hap_16 | . | . | . | . | . | . | . | - | . | … | . | . | . | . | . | . | . | . | . | . | . | . | . | . | . | . | . | . | . | . | . | . | . | . | . | . | C | . | . |
| Hap_17 | . | . | . | . | . | . | . | - | . | … | . | . | . | . | . | . | . | . | . | . | . | . | . | . | . | . | . | . | . | . | . | . | . | . | . | . | . | . | . |
| Hap_18 | . | . | . | . | . | . | . | . | . | … | . | . | . | . | . | G | . | A | . | . | . | . | . | . | . | . | . | . | . | . | . | . | . | . | . | . | . | . | . |
| Hap_19 | . | . | . | . | . | A | . | . | . | … | . | . | . | . | . | . | . | . | . | . | . | . | . | . | . | . | . | C | . | . | . | . | . | . | . | . | . | . | . |
| Hap_20 | . | . | . | . | . | . | A | . | . | … | . | . | . | . | . | . | . | . | . | . | . | . | . | . | . | . | . | . | . | . | . | . | . | . | . | . | . | . | . |
| Hap_21 | . | . | . | . | . | . | . | . | . | … | . | . | A | . | . | . | . | . | . | . | . | . | . | . | . | . | . | . | . | . | . | . | . | . | . | . | . | . | . |
| Hap_22 | . | . | . | . | . | . | . | . | . | … | . | . | . | . | . | . | . | . | . | . | A | . | . | . | . | . | . | . | . | . | . | . | . | . | . | . | . | . | . |
| Hap_23 | . | . | . | . | . | . | . | . | . | … | . | . | . | . | . | . | . | . | . | . | . | C | G | . | . | . | . | . | . | . | . | . | . | . | . | . | . | . | . |
| Hap_24 | . | . | . | C | . | . | . | . | . | … | . | . | . | . | . | . | . | . | . | . | . | . | - | . | . | . | . | . | . | . | . | . | . | . | . | . | . | . | . |
| Hap_25 | T | . | . | . | . | . | . | . | . | … | . | . | . | . | . | . | . | . | . | . | . | . | . | . | . | . | . | . | . | . | . | . | . | A | . | . | . | . | . |
| Hap_26 | . | . | . | . | . | . | . | . | . | … | . | . | . | . | G | . | . | . | . | . | . | . | . | . | . | . | . | . | . | . | . | . | . | . | . | . | . | . | . |
| Hap_27 | . | . | . | . | . | . | . | . | . | … | . | . | . | . | . | . | . | . | . | A | . | . | . | . | . | . | . | . | . | . | . | . | . | . | . | . | . | . | . |
| Hap_28 | . | . | . | . | . | . | . | . | . | … | C | . | . | . | . | . | . | . | . | . | . | . | . | . | T | . | . | . | C | . | . | . | . | . | . | . | . | . | T |
