# Supplementary material for: Intra-individual heteroplasmy in the Gentiana tongolensis plastid genome (Gentianaceae)
Source: PeerJ. 2019 Nov 27;7:e8025. doi: 10.7717/peerj.8025 (PMC6884991; doi:10.7717/peerj.8025)
Supplement: Supplemental Information 5 — Dots represent the first nucleotide in each column. Dashes represent deletion. Results of the five individuals are represented in blue, purple, black, green and red color, respectively. The individual used for Illumina sequencing is the red one. The nucleotide variation that appears in both Illumina and Sanger sequencing is bold. [file peerj-07-8025-s005.docx]

| Position | 23031 | 23062 | 23068 | 23083 | 23135 | 23160 | 23191 | 23202 | 23237 | 23253 | 23313 | 23334 | 23341 | 23349 | **23411** | 23431 | 23438 | 23445 | 23467 | 23505 | 23623 |
| --- | --- | --- | --- | --- | --- | --- | --- | --- | --- | --- | --- | --- | --- | --- | --- | --- | --- | --- | --- | --- | --- |
| Plastome | A | G | C | C | A | C | C | A | A | T | T | A | A | C | **C** | A | A | A | T | A | T |
| Hap_1 | . | . | . | . | . | . | T | . | . | . | . | . | . | . | . | . | . | . | . | . | . |
| Hap_2 | . | . | . | T | . | . | . | . | . | . | . | . | . | . | . | G | . | . | . | C | . |
| Hap_3 | . | . | . | . | . | T | . | . | . | . | . | . | . | . | . | . | . | . | . | . | . |
| Hap_4 | . | . | . | . | . | . | . | . | . | C | . | . | . | . | . | . | . | . | . | . | . |
| Hap_5 | . | . | . | . | . | . | . | . | . | . | . | . | . | . | . | . | . | . | C | . | . |
| Hap_6 | . | . | . | . | . | . | . | . | . | . | . | . | . | . | **T** | . | . | . | . | . | . |
| Hap_7 | . | . | . | . | G | . | . | . | . | . | . | . | . | . | . | . | . | . | . | . | . |
| Hap_8 | . | . | . | . | . | . | . | . | . | . | . | G | . | . | . | . | . | . | . | . | . |
| Hap_9 | . | . | . | . | . | . | . | . | . | . | . | . | . | . | . | . | . | . | . | . | C |
| Hap_10 | . | . | . | . | . | . | . | . | . | . | . | . | . | T | . | . | . | . | . | . | . |
| Hap_11 | . | . | . | . | . | . | . | . | . | . | C | . | . | . | . | . | . | . | . | . | . |
| Hap_13 | - | A | . | . | . | . | . | . | . | . | . | . | . | . | . | . | . | . | . | . | . |
| Hap_14 | . | . | - | . | . | . | . | . | . | . | . | . | . | . | . | . | T | . | . | . | . |
| Hap_15 | . | . | . | . | . | . | . | G | G | . | . | . | G | . | . | . | . | G | . | . | . |
