# Supplementary material for: Intra-individual heteroplasmy in the Gentiana tongolensis plastid genome (Gentianaceae)
Source: PeerJ. 2019 Nov 27;7:e8025. doi: 10.7717/peerj.8025 (PMC6884991; doi:10.7717/peerj.8025)
Supplement: Supplemental Information 6 — Dots represent the first nucleotide in each column. Dashes represent deletion. The individual is the one used for Illumina sequencing. The nucleotide variation that appears in both Illumina and Sanger sequencing is bold. [file peerj-07-8025-s006.docx]

| Location | 68319 | 68343 | 68344 | 68658 | 68717 | **68770** | 68868 |
| --- | --- | --- | --- | --- | --- | --- | --- |
| Plastome | CTGGCAT | - | TTTTT | A | T | **ATTGAATT** | T |
| Hap_1 | ……. | T | ….. | . | . | …….. | . |
| Hap_2 | ……. | . | ----- | . | . | …….. | . |
| Hap_4 | ……. | . | ….. | . | A | …….. | . |
| Hap_5 | ------- | . | ….. | . | . | …….. | . |
| Hap_6 | ……. | . | ….. | . | . | …….. | - |
| Hap_7 | ……. | . | ….. | G | . | …….. | . |
| Hap_8 | ……. | . | ….. | . | . | -------- | . |
